# Serologic Prevalence of *Toxoplasma gondii* in Indian Women of Child Bearing Age and Effects of Social and Environmental Factors

**DOI:** 10.1371/journal.pntd.0002737

**Published:** 2014-03-27

**Authors:** Sarman Singh, Arshi Munawwar, Sugandhi Rao, Sanjay Mehta, Naba Kumar Hazarika

**Affiliations:** 1 Division of Clinical Microbiology & Molecular Medicine, Department of Laboratory Medicine, All India Institute of Medical Sciences, New Delhi, India; 2 Kasturba Medical College, Manipal, Karnataka, India; 3 CUS Medical College, Surendra Nagar, Gujarat, India; 4 Assam Medical College, Gauhati, Assam, India; University of Pittsburgh, United States of America

## Abstract

**Background:**

Seroprevalence and incidence of toxoplasmosis in women of child bearing age has remained a contentious issue in the Indian subcontinent. Different laboratories have used different patient recruitment criteria, methods and variable results, making these data difficult to compare.

**Aim:**

To map the point-prevalence and incidence of toxoplasmosis in India.

**Material and Methods:**

In this cross-sectional study, a total of 1464 women of fertile age were recruited from 4 regions using similar recruitment plans. This included women from northern (203), southern (512), eastern (250) and western (501) regions of India. All samples were transported to a central laboratory in Delhi and tested using VIDAS technology. Their age, parity, eating habits and other demographic and clinical details were noted.

**Results:**

Most women were in the 18–25 years age group (48.3%), followed by 26–30 years (28.2%) and 31–35 years (13.66). Few (45) women older than 35 yr. were included. Overall prevalence of anti-Toxoplasma IgG antibodies was seen in 22.40%, with significantly more in married women (25.8%) as compared to single women (4.3%). Prevalence increased steadily with age: 18.1% in the 18–25 yr. age group to 40.5% in women older than 40 yr. The prevalence was high (66%) in those who resided in mud houses. Region-wise, the highest prevalence was observed in South India (37.3%) and the lowest (8.8%) in West Indian women. This difference was highly significant (P<0.001). Prevalence was 21.2% in East India and 19.7% in North India. The IgM positivity rate ranged from 0.4% to 2.9% in four study centers.

**Conclusions:**

This pan-India study shows a prevalence rate of 22.4% with a wide variation in four geographical regions ranging from as low as 8.8% to as high as 37.3%. The overall IgM positivity rate was 1.43%, indicating that an estimated 56,737–176,882 children per year are born in India with a possible risk of congenital toxoplasmosis.

## Author Summary

Toxoplasmosis is a protozoan parasitic disease commonly transmitted and propagated by cats as family pets. Infection acquired during pregnancy can lead to congenital abnormalities in the fetus, still birth or intrauterine death. Seroprevalence and incidence of toxoplasmosis in Indian women of child bearing age has remained a contentious issue. Different laboratories have used different patient recruitment criteria, methods and variable results, making these data unreliable. There is no published pan-India seroprevalence study. Hence, a seroprevalence study was undertaken comprising 1464 women of reproductive age representing four distinct geographical regions of India. This resulted in an estimated prevalence of 22.4% (328); the highest prevalence being in South India (37.3%) followed by East India (21.2%) and North India (19.7%). West Indian women had the lowest seroprevalence (8.8%). This difference was highly significant. In our analysis we determined the possible risk-factors of infection in these women. These included lower socioeconomic status, residing in mud plastered houses, consumption of raw salad, drinking untreated water, owning pets and advanced age. Overall, the incidence rate of toxoplasmosis was 1.43%. Extrapolating the data, we estimate that between 56,737 and 176,882 children a year may be born in India with a possible risk of congenital toxoplasmosis, which can manifest itself in-utero or several years after birth.

## Introduction

*Toxoplasma gondii* (*T. gondii*) infection is a significant member of the TORCH group of diseases which cause congenital abnormalities, and even fetal loss. TORCH group infectious agents also consist of Rubella, Cytomegalovirus, Herpes viruses and *Treponema pallidum*. In India, awareness about these infections that cause congenital conditions is poor [1]–[4]. Most women who seek medical attention, or are referred by obstetricians, are those who have had an undesirable pregnancy outcome [5], [6].

Toxoplasmosis is caused by the protozoan parasite *T. gondii*. It has a wide host range, infecting most warm-blooded species but the life cycle is completed only in felids [7], [8]; Indeed only cats can shed the environmentally-resistant stage of the parasite (oocyst) in their feces [9], [10]. Humans usually become infected by ingesting food or water contaminated with cat faeces containing oocysts [11], [12] or by eating under-cooked meat containing the encysted stage of the parasite (tissue cysts) [13], [14]. Infection acquired during pregnancy can be transmitted to the fetus, sometimes with serious consequences. There are numerous serological surveys of *T. gondii* infection in pregnant women in India, but most of them were based on convenience sampling, and often selectively in women with bad outcome of pregnancy [15]–[18].

Here, we present the first designed survey for determining the prevalence rate of anti-*T. gondii* antibodies in Indian women of reproductive age from four geographic regions: East, West, North and South India.

## Methods

### Participants and sampling

#### Geo-climatic conditions of the selected study centers

We selected 4 regions of India with maximum diversity in cultural and climatic conditions to see if these environmental factors are associated with variation in the incidence and prevalence of toxoplasmosis (Table 1). Western India falls under an arid zone climate, with a maximum temperature reaching on average 46°C in May-June, while the selected population in South India resides in a humid and moderately hot environment. The climatic conditions where North and East Indian populations reside are similar to the South but their living standards and rural/urban backgrounds were significantly different. The four institutions that agreed to participate in this multicenter study are listed in table 1. To represent 4 geographical regions of India, a total of 1772 women were enrolled. Female volunteers from the hospital staff, as well as from the local community, colleges, and antenatal clinics were recruited in the study.

**Table 1:** Climatic and topographic characteristics of the four regions from where study subjects were recruited.

Geographical Region	Average rain fall (mm)	Temperature		Climate Type (Köppen climate classification)	Elevation above sea level	Coordinates	
		Minimum	Maximum			Latitude	Longitude
South India (Karnataka)	3,456	21°C	34°C	Tropical wet, humid and rain forests	120 m = 393.70 ft.	12.9702° N	77.5603° E
West India (Gujarat)	578	13°C	46°C	Sub-tropical arid, dry and hot	224 m = 734 ft.	22.7300° N	71.5100° E
East India (Assam)	2,818	10°C	35°C	Tropical wet, monsoon rainforest	91 m = 298.55 ft.	26.1400° N	91.7700° E
North India (Delhi NCR)	617	8°C	44°C	Sub- tropical, monsoon rains	213 m = 698.00 ft.	28.6100° N	77.2300° E

All participating women were administered a questionnaire (Supplementary file S1) before collection of blood sample, which provided information about their age, marital status, education, socioeconomic level, type of accommodation, eating habits, contact with pet and stray animals, exposure to soil and present/past obstetric history. Women having severe metabolic and autoimmune disorders, such as rheumatoid arthritis or immune deficiencies including AIDS, cancer and those on immune suppressive therapies and suspected of intrauterine TORCH infections, were excluded. Each woman was provided information on the disease and why it was important to know her sero-positivity status, as per our IRB guidelines (patient information sheet). Only one sample from each woman was collected. Presuming a prevalence rate of 45% in pregnant women of India [3], statistically we needed each center to collect a minimum of 400 samples. Through venipuncture, 5 mL of blood sample was collected in a sterile container and serum was separated at the participating centers. The separated serum samples were stored locally at 4°C for a maximum period of 3 days and then samples from eligible participants were sent biweekly to the central laboratory at the All India Institute of Medical Sciences (AIIMS), New Delhi through courier (DHL, India) delivery. Data from the questionnaire was entered in an excel sheet. The laboratory technician was blinded on participant clinical or socio-demographic details. The study coordinator (SS) evaluated the exclusion and inclusion criteria. AIIMS being the tertiary care and federally funded hospital (north India), all samples collected at this center were tested as routine patient care, irrespective of their eligibility criteria for the study. However, for the present study only 203 women from North India were selected (see flow chart).

### Ethical aspects

Blood sampling was performed from October 2011 to October 2012. The Institutional Review Board of AIIMS approved this study (IEC/NP-92/2011). Informed written consent was obtained from all participating women who agreed to participate in the study. The study followed the STROBE guidelines (Supplementary file S2).

### Anti-*Toxoplasma* IgG and IgM antibody detection assays

Serum samples were assayed for anti-*Toxoplasma* IgG and IgM antibodies by a commercially available Vitek Immuno-Diagnostic Assay System (VIDAS, BioMerieux SA, France), strictly following the manufacturer's instructions. All IgG and IgM positive samples were tested for IgG avidity using the same technology (VIDAS, BioMerieux SA, France), as published previously [3]. An avidity index of <0.200 indicates low avidity; an index of 0.200–0.299 indicates borderline avidity, and an index of >0.300 denotes high avidity for IgG. High avidity enables exclusion of a recent infection of <4 months duration. More details are provided in a related publication [3].

### Statistical analysis

Data was entered in Excel sheet and imported into SPSS statistical program for analysis. For statistical evaluation of binomial data, the χ^2^ test with 95% confidence intervals according to Clopper and Pearson were used; P values <0.05 were considered statistically significant. Incidences (IgM positivity) and prevalence (IgG positivity) rates are expressed as percentages. To estimate the approximate number of babies born with risk of congenital *T. gondii* infection in India per annum, following formula was used. 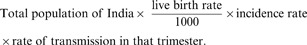



Total population and live birth rates were taken from the Government of India official website [19].

For example, we had 434 women in their first trimester, of whom 7 (1.61%) were IgM positive. In the first trimester of pregnancy the rate of congenital transmission is reported to be 13% [6]. Hence, presuming that all women were in their first trimester, the approximate number of children born with a risk of congenital *T. gondii* infection would be 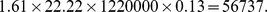



Using the same formula, out of 177 women in their second trimester, 4 (2.25%) women were IgM positive. Hence presuming a transmission rate of 29% in the second trimester [6], the approximate number of children born with a risk of congenital *T. gondii* infection would be 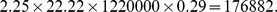



## Results

### Age distribution and obstetric details of the subjects

**Figure 1:**
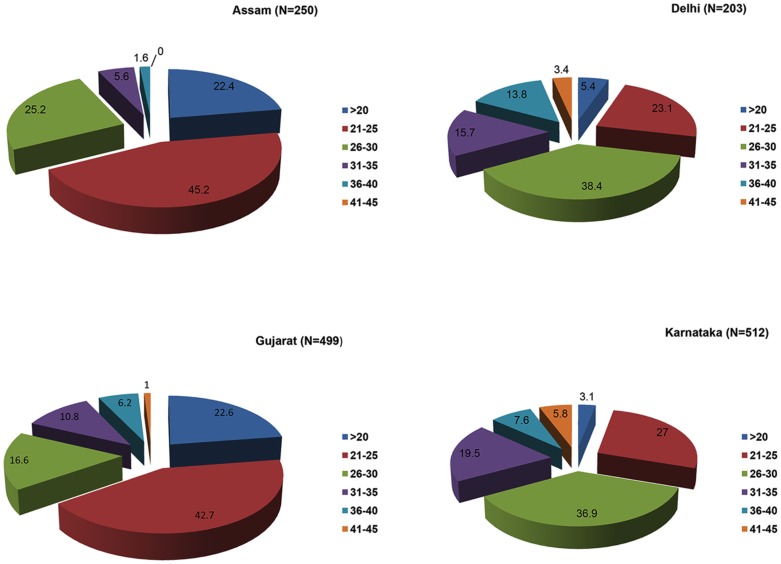
Distribution (%) of women in different age groups from 4 regional study centers. Maximum number of women who volunteered for the inclusion in the study were in their twenties and early thirties.

**Figure 2:**
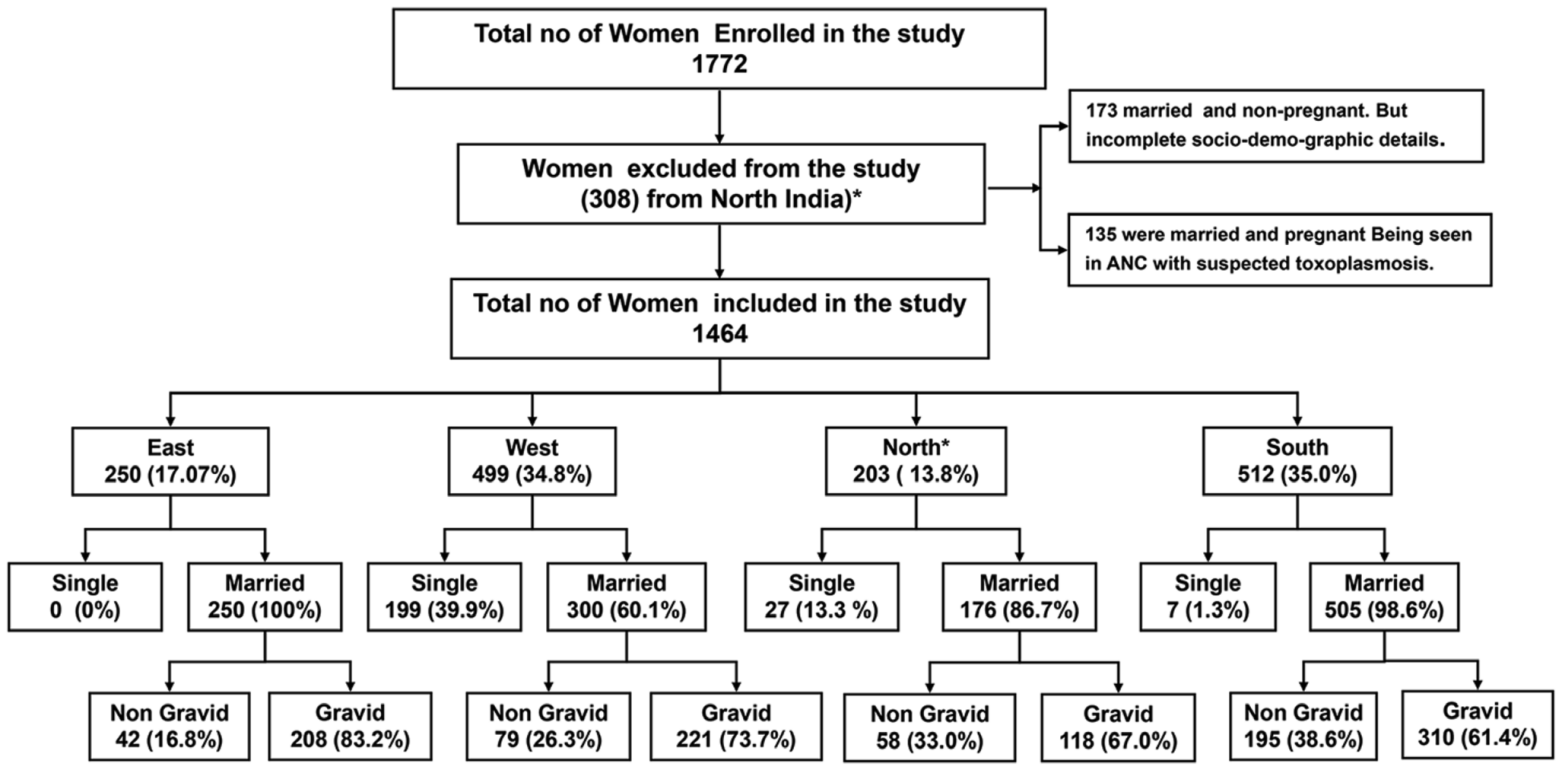
Flow chart showing women included from each center with details of their marital and pregnancy status.

Data on 1464 women of reproductive age which ranged from 18 to 45 years (mean ± SD, 26.9±5.9) from four geographically distinct regions of India are presented here. Of these, 250 (17.1%) were from East India (Assam), 203 (13.8%) from North India (Delhi and national capital region), 499 (34.1%) from Western India (Gujarat), and 512 (34.9%) from South India (Karnataka). Region-wise, the mean age of the women in the East was 24.2±4.2 yr., 29.3±5.8 yr. in the North, 25.2±5.9 yr. in the West and 29.2±5.7 yr. in the South. The difference was insignificant. The distribution of various age groups is shown in figure 1. Out of 1464 women, 233 (15.9%) were single with a mean age of 22.4±3.8 (18–41 yr. age range) while 1231 (84.08%) were married with a mean age of 27.8±5.9 (18–45 yr. age range). Of the 1231 married women, 297 (24.1%) were nulliparous with a mean age of 30.2±6.3 and 934 (75.9%) were parous with a mean age of 27.1±5.6 (Figure 2, Flow chart 1). All single women were nulliparous and non-pregnant. Of the 934 parous women, 471 (50.4%) were single gravida and 463 (49.6%) were multigravida. Their mean age was 25.3±5.0 and 28.8±5.6, respectively. The number of gravida was as high as 7 (6 women; 1.3%). Of the 934 gravida women 356 (38.2%) had at least one live birth, while 153 (16.4%) had all adverse pregnancy outcomes. The remaining 424 (45.4%) were primigravida. Overall, 751 women were pregnant at the time of sample collection. Of these, 434 (57.8%) were in their first trimester, 176 (23.4%) in their second and 141 (18.8%) in their third trimester.

### Frequency of anti-*Toxoplasma* IgG and IgM antibodies

**Figure 3:**
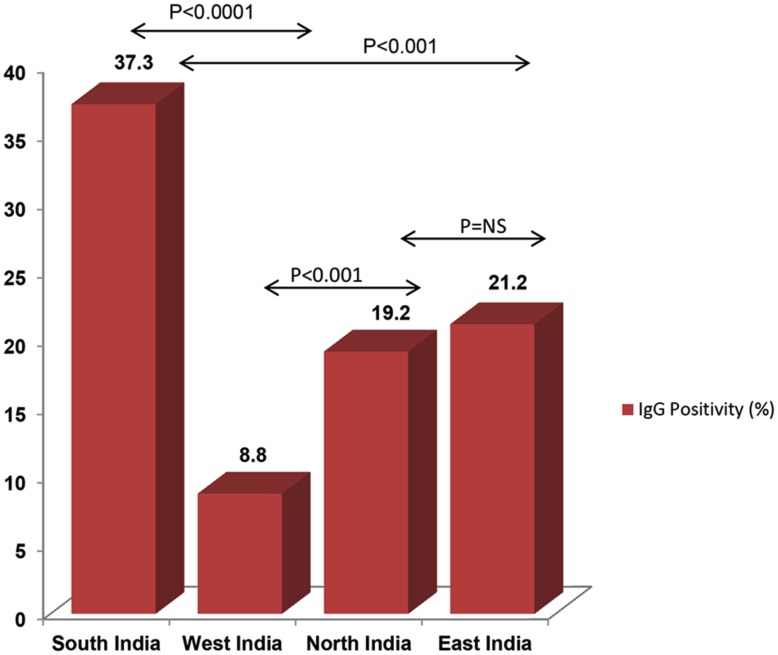
Prevalence of toxoplasmosis in women from 4 regional study centers. The prevalence rates varied from one region to another but the most significant difference was between South India and the other three regions.

**Figure 4:**
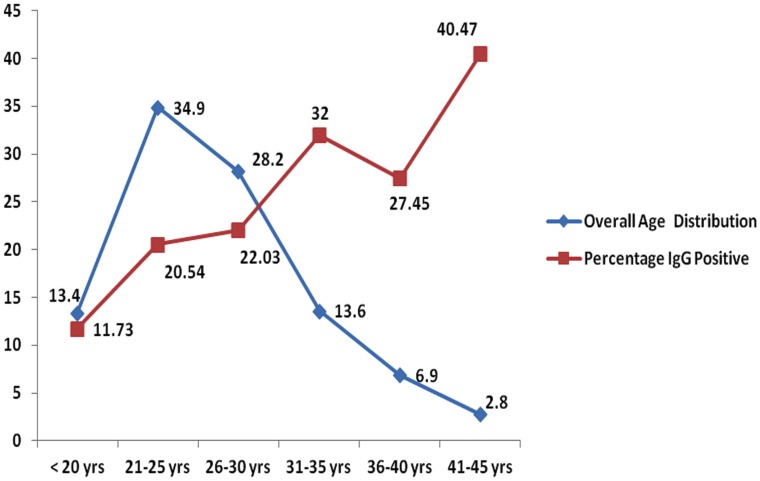
Age distribution of eligible women and relation of age with prevalence of toxoplasmosis. The prevalence increased with age of subjects even if their total number decreased.

**Figure 5:**
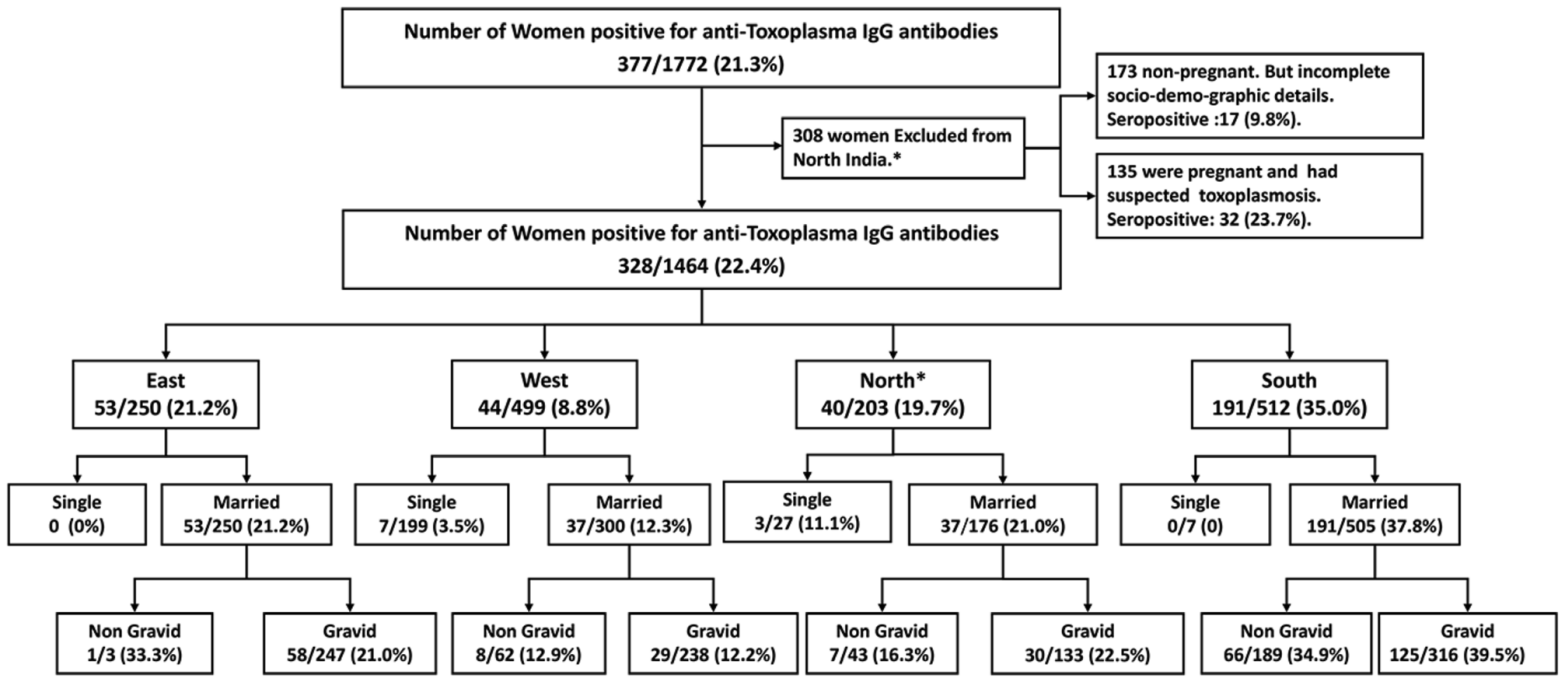
Seroprevalence of toxoplasmosis in unmarried, married and in those who were gravida and non-gravida women. These prevalence rates are shown per region.

The overall seroprevalence was 22.4% (328 of 1464). The prevalence rates varied significantly across the 4 regions, with the highest (37.3%) in South India and the lowest in West India (8.8%). The difference was highly significant (Figure 3). We also observed a significant difference in the prevalence rates of anti-*Toxoplasma* antibodies between single women (4.29%, 95% CI; a range of 1.9% to 6.9%) and married women (25.0%, 95% CI; a range of 22.6% to 27.4%) (p<0.005). However, seroprevalence increased with age (Figure 4, trend line). Prevalence was lower than 11.7% among those under 20 yr. of age, but steadily increased to 40.4% in those who were older than 41 yr. Region-wise proportion of anti-*Toxoplasma* IgG antibody positive women in various age groups is shown in table 2. Most (74.2%) of the 328 seropositive pregnant women were multigravida and only a quarter (25.8%) were primigravida (Figure 5, Flow chart 2). Of the 208 seropositive pregnant women at the time of sampling, two thirds (138/208 or 66.35%) were in their first trimester while 36/208 (17.30%) were in their second trimester and 34/208 (16.35%) in their third.

**Table 2:** Seroprevalence of toxoplasmosis in women of 4 geographical regions and their age groups.

Age Group ---->	≤20 yr	21–25 yr	26–30 yr	31–35 yr	36–40 yr	41–45 yr	Total
East India (Assam) N = 250	56 (22.4)	113 (45.2)	63 (25.2)	14 (5.6)	4 (1.6)	0	250 (21.2)
West India (Gujarat) N = 499	113 (22.6)	213 (42.7)	83 (16.6)	54 (10.8)	31 (6.2)	5 (1)	499 (8.8)
North India (Delhi NCR) N = 203	11 (5.4)	47 (23.1)	78 (38.4)	32 (15.7)	28 (13.8)	7 (3.45)	203 (19.7)
South India (Karnataka) N = 512	16 (3.13)	138 (26.9)	189 (36.9)	100 (19.5)	39 (7.62)	30 (5.8)	512 (37.3)
Total	196	511	413	200	102	42	1464
Seropositive (%)	23 (11.7)	105 (20.5)	91 (22.0)	64 (32.0)	28 (27.4)	17 (40.4)	328 (22.4)

Only 21 women out of 1464 (1. 43%) had anti-*Toxoplasma* IgM antibodies. Trimester-wise, 434 women were in their first trimester and 7 (1.61%) of them were IgM positive, while 177 women were in their second trimester and 4 (2.25%) of these had IgM antibodies. None of the 141 pregnant women in their third trimester was IgM positive. Ten of 479 non-pregnant women had IgM antibodies. IgM positivity was highest (2.9%, 15 out of 512) in South India, followed by 0.8% in Eastern (2 out of 250), 0.6% in Western (3 of 499) and 0.4%, in North (1 out of 203) India. The region-wise pattern was similar to the prevalence rate of IgG antibodies. All IgM positive women were also IgG positive and none, except for one woman, was IgG negative and IgM positive. From South India, 191 women were seropositive, of which 15 (7.8%) were IgM positive, while in North India only 1 out of 40 (2.5%) and in East India 2 out of 53 (3.7%) IgG positive women were also IgM positive. However, in West India the IgM positivity rate amongst the IgG positives was 6.8% (3 out of 44). This change was significant (<0.001) when estimated out of the total study subjects as opposed to only out of IgG positives. Out of 21 IgM positive subjects, 6 had low avidity, 8 were borderline and 7 showed high avidity. All low avidity subjects were followed up for 6 months. Of these, 3 were pregnant and 2 delivered normal babies while one newborn had congenital hydrocephalus and microphthalmia. Follow-up samples from babies could not be tested. All low avidity seropositive women were from South India.

### Socio-demographic characteristics and of the study subjects

Most women (97.1%) belonged to low or lower middle income groups. The majority of women from South and East India resided in mud-plastered houses and consumed tube well/hand pump water without using any disinfectant or filter. The difference in the living conditions was highly significant (p<0.0001) between South India and West India and also between South India and North India (p<0.001), while the difference between North and East India was not significant (Figure 3). General socio-behavioral characteristics of these women are shown in table 3. Most women (1253 of 1464; 85.6%) were involved in housekeeping. In spite of having a rural background (75.1%), our study showed that 90.6% (1328) of the women had elementary education. A history of contact with animals was found in 426 (29.1%) women but pets were significantly more common (53.5%) in South Indian households (Table 3). Consumption of raw salad was common (1360/1464; 92.89%) across the country. Of the 308 women from North India who are excluded from our final data analysis, 178 were parous and 130 were nulliparous. Their mean age was 29.1 yr. (range of 15–52 yr.). Of the parous women, 78 (43.8%) were primigravida and 45 (25.3%) were two gravida. Fifty two (29.2%) women were multigravida and the number of gravida was as many as eight.

**Table 3:** Socio-demographic and other probable risk factors for acquiring toxoplasmosis in women from different regions of India.

Region of India	N =	Low/Lower middle Socio-economic status	Reside in Mud house	Reside in Cemented house	Consume Raw salad	Drink Tap water	Drink Tube well/Hand pump water	Own Pet	Own Cat	IgG prevalence (%)
East	250	250	118	132	241	68	182	59	0	53
		(100)	(47.2)	(52.8)	(96.4)	(27.2)	(72.8)	(23.60)		(21.2)
North	203	201	11	192	183	179	24	42	7	40
		(99.0)	(5.4)	(94.6)	(90.1)	(88.2)	(11.8)	(20.7)	(3.4)	(19.7)
West	499	469	99	400	415	383	116	51	6	44
		(94.0)	(19.8)	(80.2)	(83.2)	(76.7)	(23.3)	(10.2)	(1.2)	(8.8)
South	512	501	307	205	501	182	330	274	166	191
		(97.8)	(60.0)	(40.0)	(97.8)	(35.5)	(64.5)	(53.5)	(92.7)	(37.3)
Total	1464	1421	535	929	1340	812	652	426	179	328
		97.0	36.5	63.5	91.5	55.5	44.5	29.1	12.2	(22.4)

Occurrences (%).

## Discussion

This community based study provides a significant and much needed resource material specific to India. Our study showed that age was one major variable of a higher prevalence rate of toxoplasmosis. The prevalence was also higher in those who were married and multigravida than those who were unmarried/single. This higher prevalence was not associated with the number of gravida *per se* but rather to higher mean age [27.8±5.9 (range 18–45 yr.)] of those who were married than those who were unmarried/single women [22.4±3.8 (range 18–41 yr.)]. This conclusion is further validated with observations that age of women from South India was higher than other regions and prevalence was also significantly higher in women of this region. There are also anecdotal reports associating multiple sexual exposures with high prevalence and possibility of sexual transmission of toxoplasmosis [20]. Over all prevalence of toxoplasmosis in the present study was significantly lower than reported earlier from India [3]. One plausible explanation for this difference could be that in the present study we included all women of fertile age from various cohorts, as compared to an earlier study in which we included only pregnant women. However, even if we combine those with suspected TORCH infections, who were otherwise excluded from final data analysis, the overall prevalence rate in North Indian women was 17.4%, which was significantly lower than the 45% reported by us 10 years ago, in the same population using the same diagnostic techniques. Whether it was due to improved awareness and social hygiene in the last 10 years, or due to selection biases in the two studies, cannot be ascertained with certainty.

The difference in the prevalence rate between women from South and West India was highly significant. It could be mainly due to socio-cultural and climatic factors. The climatic conditions in South India favour sustenance and proliferation of *Toxoplasma* oocysts. Also a highly significant number of households owned cats in this region. Moreover, as a social culture, South Indians do not wear shoes and most often are barefoot or wear sleepers only. This might increase chances of transferring *T. gondii* oocysts from soil and water to their food [9]–[13]. Western India, on the other hand, is a dry arid climatic zone where temperature in May-June averages 46°C. Socio-culturally also, the population in West India must wear shoes due to the high temperature and sandy soil. These climatic conditions are detrimental to *T. gondii* to maintain its life cycle. We [21] and others [22], [23] have previously demonstrated the role of environmental conditions on the prevalence of toxoplasmosis.

Although consumption of raw/undercooked meat and exposure to soil through farming or gardening have been associated with a higher risk of infection in various studies [1], [4], [21], no such correlation was observed in the multivariate analysis done in the present study. In India, consumption of raw or undercooked meat is extremely rare, hence this route of infection is theoretically negligible. The type of food, social considerations and quality of water consumed were the most likely factors associated with high prevalence of toxoplasmosis in South India, besides higher age. Water and food-borne outbreaks of toxoplasmosis have been well documented worldwide [11], [12] and also from India [24].

Anti-toxoplasma IgM antibodies were considered indicative of a recent infection for several decades, but then it was realized that these antibodies could persist for several months, even years after the primary infection [2], [3], [5]. Hence in the 1990s a new test, based on affinity levels of IgG antibodies binding with antigen, was developed, and known as the avidity test. Determination of avidity helps in determining if the infection is of recent origin or more than 4 months old [25]–[27]. The IgM positivity and low avidity rates we observed in this study may seem very low, but cumulative figures are alarming. India has a population of more than 1.22 billion and the live birth rate is 22.22/1000 per annum [19]. Taking these IgM rates into consideration, a conservative estimate of child births with a possible risk of congenital toxoplasmosis [6] would be between 56737 and 176882. This would translate into health and rehabilitation expenses to treat and rehabilitate the congenitally-infected children, many of whom may remain asymptomatic for several years. Unfortunately, many of such congenitally-infected adolescents and adults are not diagnosed accurately, whether the infection was acquired in-utero or after birth. Our calculations, which are based only on IgM positivity rates, are not a very reliable marker of recent infection, as discussed in the previous paragraph. Therefore, these estimates need more validation studies from India for transplacental transmission rates of *T. gondii* using IgG avidity or molecular methods, such as PCR. Due to a high false IgM positivity rate, we also conclude that carrying out only IgM testing without IgG testing is not an advisable approach of investigating toxoplasmosis, and all patients must be tested first for IgG and, if found positive, samples would be subjected to IgM and/or avidity tests.

Our study had some limitations. We attempted to extract a maximum of information regarding pan-India seroprevalence of toxoplasmosis and correlate it with environmental and socio-cultural considerations. It would have been ideal to include more centers and more samples but this was not feasible due to time and financial constraints. Also, we could not do multiple follow-up sampling to find out true incidence rates in various seasons and in IgM positive women.

## Supporting Information

Supplementary File S1 Patient questionnaire administered to the subjects.
(PDF)

Supplementary File S2 Strobe checklist.
(DOCX)
