# Supplementary material for: Serologic Prevalence of Toxoplasma gondii in Indian Women of Child Bearing Age and Effects of Social and Environmental Factors
Source: PLoS Negl Trop Dis. 2014 Mar 27;8(3):e2737. doi: 10.1371/journal.pntd.0002737 (PMC3967963; doi:10.1371/journal.pntd.0002737)
Supplement: Supplementary File S1 — Patient questionnaire administered to the subjects. (PDF) [file pntd.0002737.s001.pdf]

Centre code: 

# Clinical Investigation Proforma

# Sero-epidemiology of toxoplasmosis in women of child bearing age in India

**Phase of study: - Epidemiological study**

Abbott
A Promise for Life

Date_____/_____/_______

Participant ID   --------------------------------------

If attending any hospital:  Reg. No. ---------------------OPD------------------Medical Reason-----------------------------

Name ___________________________          Age (Yrs) _____

Father/Husband's name___________________________

Address___________________________________________________City______________State___________

Contact No (s)______________________________________________________________________________

Marital status :  Single ☐  Married ☐

**Education**

Illiterate ☐  Matric ☐  Graduate ☐  Post Graduate ☐  Doctorate/ Professional course ☐

Occupation: - Yes ☐  No ☐

If Yes kindly mention: _________________________________________

**Socio-Economic Level**

Low ☐  Medium ☐  High ☐

**Eating Habits:**

**Vegetarian** ☐  **Non-Vegetarian** ☐

**If Non-Vegetarian:**

Beef ☐  Mutton ☐  Pork ☐  Poultry ☐  Fish ☐

Frequency of consumption of meat: ________________________

**Accommodation:**

Pucca ☐ Kachcha ☐ Mud hut ☐ Tent ☐

**Pets:** No ☐ Yes ☐

| Type of animal | Exposure Date | Exposure Location | City / State |
|---|---|---|---|
| ☐ Bird | | | |
| ☐ Cat | | | |
| ☐ Dog | | | |
| Other pet Specify ______________ | | | |

**Exposure Details:**

Farming ☐ Gardening with bare hand/foot ☐ Eating raw salads ☐ Raw meat ☐

Regarding Raw salad, please specify:

Carrot ☐ Radish ☐ Beet Root ☐ Cauliflower ☐ Celery ☐ Cabbage ☐ Cucumber ☐

**Drinking Water Sources:**

Tap Water ☐ Hand pump ☐ Tube well/well ☐ Spring water ☐ Filtered ☐ River Water ☐

**Drinking Milk**

Boiled ☐ Pasteurized ☐

**Obstetric history:**

If Married: Non-Pregnant ☐ Pregnant ☐ LMP:__________________

**HIV status: Positive** ☐ **Negative** ☐ **Duration________**

**Corticosteroids: Yes** ☐ **NO** ☐

**History of Allergy/Allergens: Yes** ☐ **NO** ☐ **Type ___________**

**Trimester of Pregenancy**

**First** ☐ **Second** ☐ **Third** ☐

| Gravida status | Alive (Healthy/Sick)<br>Please write details | Male/ Female/Aborted | If aborted<br>SB/ SA/MTP |
|---|---|---|---|
| G1 | | | |
| G2 | | | |
| G3 | | | |
| G4 | | | |
| G5 | | | |
| G6 | | | |

**Laboratory Details**

- Sample collected on (dd/mm/yy) : ……..…/………/……………
- Sample sent to Department of Clinical Microbiology, AIIMS Delhi on (dd/mm/yy):……..…/………/……………

***Investigators Signature with date***

**FOR AIIMS USE ONLY**

- Sample Received at DEPARTMENT OF Clinical Microbiology, AIIMS Delhi on (dd/mm/yy) :

  …….…..…/………/……………

- Test Performed at Department of Clinical Microbiology AIIMS on (dd/mm/yy) :

  ……..…/…..……/……………

**Test Results:**

| Test | Cut-off value ( give lot no. of the kit) | Test value | Interpretation | Signatures of technician |
|---|---|---|---|---|
| IgG | | | | |
| IgM | | | | |
| IgG- avidity | | | | |

***Authentication with date***

**Prof. Sarman Singh**
Division of Clinical Microbiology,
Department of Laboratory Medicine
All India Institute of Medical Sciences
Ansari Nagar
New Delhi
